# The Development of a New Vagus Nerve Simulation Electroceutical to Improve the Signal Attenuation in a Living Implant Environment

**DOI:** 10.3390/s24103172

**Published:** 2024-05-16

**Authors:** Daeil Jo, Hyunung Lee, Youlim Jang, Paul Oh, Yongjin Kwon

**Affiliations:** 1 Department of Industrial Engineering, Ajou University, Suwon 16499, Republic of Korea; mynamedaeil@ajou.ac.kr; 2 Oceans Bio Co., Ltd., Seoul 04303, Republic of Korea; leehu@oceansbio.com (H.L.); yrjang@oceansbio.com (Y.J.); 3 Department of Mechanical Engineering, University of Nevada-Las Vegas, Las Vegas, NV 89154, USA; paul.oh@unlv.edu

**Keywords:** electroceutical, VNS, iVNS, IPG, implantable pulse generator, VNS stimulation therapy

## Abstract

An electroceutical is a medical device that uses electrical signals to control biological functions. It can be inserted into the human body as an implant and has several crucial advantages over conventional medicines for certain diseases. This research develops a new vagus nerve simulation (VNS) electroceutical through an innovative approach to overcome the communication limitations of existing devices. A phased array antenna with a better communication performance was developed and applied to the electroceutical prototype. In order to effectively respond to changes in communication signals, we developed the steering algorithm and firmware, and designed the smart communication protocol that operates at a low power that is safe for the patients. This protocol is intended to improve a communication sensitivity related to the transmission and reception distance. Based on this technical approach, the heightened effectiveness and safety of the prototype have been ascertained, with the actual clinical tests using live animals. We confirmed the signal attenuation performance to be excellent, and a smooth communication was achieved even at a distance of 7 m. The prototype showed a much wider communication range than any other existing products. Through this, it is conceivable that various problems due to space constraints can be resolved, hence presenting many benefits to the patients whose last resort to the disease is the VNS electroceutical.

## 1. Introduction

An electroceutical is a medical device that uses electrical signals to control biological functions. Electroceuticals aim to treat various diseases and conditions by using the body’s electrical properties. Unlike the traditional drugs, it does not use chemicals but instead regulates the functions of the nerve system, muscles, and other tissues through electrical stimulation. Electroceuticals are used in a variety of medical fields and are effective in treating various conditions, including chronic pain, heart disease, diabetes, depression, and neurological disorders. Electroceuticals are commonly inserted into the body in the form of implants, but they can also be worn externally on the body. Electroceuticals provide a new and innovative treatment method that can improve the quality of life for patients, especially in cases where traditional drug treatment is either ineffective or impaired with side effects. The electroceutical field continues to grow and is expected to play an important role in the future of medical technology [1,2,3,4].

Currently, electroceuticals are mainly divided into vagus nerve stimulation (VNS), deep brain stimulation (DBS), and spinal cord stimulation (SCS), as shown in Table 1. The human nerve system includes the central nerve system and the peripheral nerve system, which is derived from the central nerve system. This system distributes branches throughout the human body and transmits nerve signals to organs [1,2]. When the function of the brain’s nerve system is damaged, various neurological diseases may occur such as stroke, depression, epilepsy, and Parkinson’s disease. It is possible to restore the damaged functions with artificial nerve stimulation. This type of treatment is called neuromodulation, and some of its effects have been proven. The treatments such as DBS and VNS have been approved by the FDA and are currently being used for their intended purpose [5,6,7].

**Figure 1:**
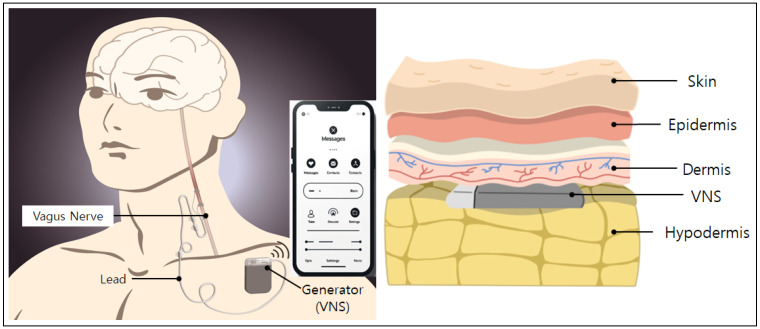
Brief illustration of human implantable VNS.

DBS is a method of providing direct electrical stimulation to specific areas of the brain. It is primarily used for treating neurological disorders such as Parkinson’s disease, obsessive-compulsive disorder, and major depression. Small electrodes are implanted inside the brain, and precise electrical stimulation is delivered through a battery-operated transmitter to regulate the brain activities. SCS is a treatment method that blocks or modulates the transmission of pain signals by providing electrical stimulation to the spinal cord. It is used to manage conditions such as chronic pain, complex regional pain syndrome (CRPS), and post-operative pain. Electrodes are placed near the spinal cord and stimulation is provided through a transmitter implanted under the skin. This stimulation relieves the patient by changing the way they feel the pain. VNS is mainly indicated for drug resistant epilepsies. The electrical stimulation device is inserted between the subcutaneous tissue and the dermis layer in the patient’s chest. It includes a programming device for wire control and charging and an auxiliary control device for the patient (Figure 1). The implant surgery requires making an incision in the armpit skin, inserting an electrical signal generator subcutaneously, and connecting the electric signal generator to the vagus nerve in the neck area [8].

Approximately 10 million people worldwide and 10,000 people in South Korea suffer from drug-refractory epilepsies, making up 30% of all epilepsy patients. When there is no more improvement after multiple drug prescriptions (be it cocktails or new drugs), a surgery can be indicated (temporal, extratemporal resections, or callosotomy). However, if the surgery is not indicated or is ineffective, VNS can be proposed. The implantable electroceutical requires a short-distance communication for data communication. Short-distance communication has a licensed frequency band of 402 to 500 MHz. This is called the medical implant communication service (MICS), which is a limited frequency band allocated for the special purpose of electroweak. The use of this limited band causes great difficulties in selecting and supplying components for various electroceutical applications, and has emerged as a major problem in the research and development process. On the other hand, a 2.4 GHz frequency has the advantage of having a wide selection of processors and components that are readily available. Even though unlicensed for electroceuticals, this expands the usability and has the advantage of being easy to link with mobile devices that are already a part of people’s daily life. It is also in line with the recent trends such as none-face-to-face medical care and the Internet of Things (IOT)-based patient monitoring. However, for the 2.4 GHz frequency, the skin penetration depth is only about 7 mm, meaning that there is a possibility of communication failure when implanted in large animals or human bodies. Currently, the number of devices adopting unlicensed 2.4 GHz bands, especially with the Bluetooth protocol, is increasing rapidly on a global scale. As shown in Table 2, the global implantable electroceutical market is trending towards adopting Bluetooth as a communication protocol. This is due to its versatility, appropriate communication strength, and ease of supply for parts. Close examination reveals that four out of seven electroceutials that received the FCC certification since 2020 have implemented wireless communication within the human body using the Bluetooth Low Energy (BLE) protocol (i.e., 2.45 GHz). The FCC standard (KDB 447498 D01 General RF Exposure Guidance v06 [9]) limits the level of radio emissions as well as the conduction of implantable medical devices to be less than 1 mW. This also complies with the South Korean standard. Since most implantable electroceuticals must control the device, monitor the patient’s status, and exchange various information collected from a patient using wireless communication, the importance of wireless communication technology is increasing. In addition, the development needs for advanced technologies, such as IoT-based VNS, wide range energy transmission devices, and high-output beamforming, are getting ever more important within the domain of implantable electroceutical [9,10].

**Figure 2:**
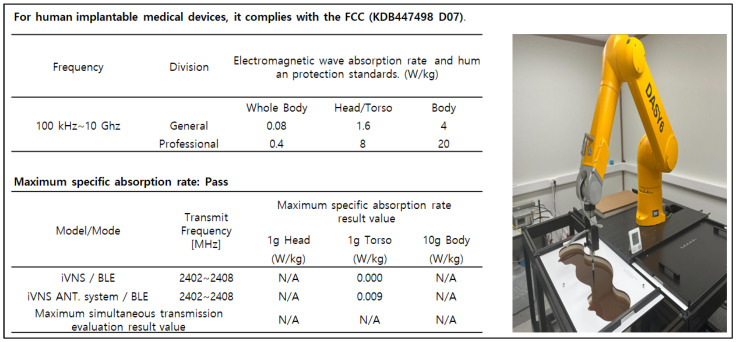
Human absorption rate verification. In relation to human hazards, implantable medical devices using Bluetooth must meet FCC standard KDB447498 D07 [9], with output standards of less than 1 mW per unit time for electroceutical and less than 7.5 mW for transmitters. To verify this, specific absorption rate (SAR) was measured by an accredited agency and judged suitable.

**Figure 3:**
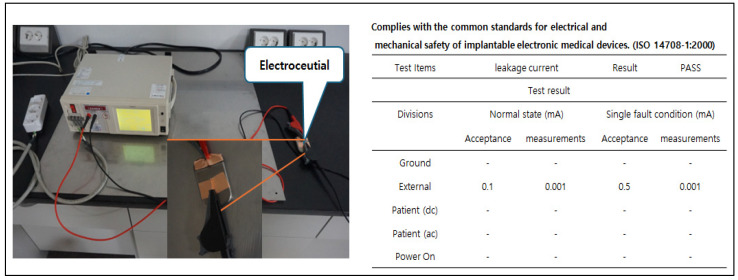
Current leakage verification that complies with safety standards for medical devices (ISO 14708-1:2014) [12]. In accordance with common standards for electrical and mechanical safety of implantable electroceutical, leakage current should be less than 1 μA. As a result of measurement by an accredited agency, it was found to be less than 1 μA.

In relation to this study, the important aspects of VNS research are focused on the drug-resistant patients. To be more specific, the patients receive the drug treatment for epilepsy before VNS implantation. If drug resistance is found and cannot be treated anymore, the efficacy and follow-up studies of epilepsy patients after VNS implantation are initiated. For children with epilepsy, finding the efficacy and side effects after VNS implantation is a very important research issue. The problem is that there is a limited database for VNS which contains the information about the long-term effects after VNS implantation. This situation considerably limits the depth of post-VNS implantation monitoring studies. This is believed to be due to the high cost of VNS electronic devices and the procedure itself, hence preventing many epilepsy patients from benefiting from the implantation. As a result, the implantation process tends to be conducted passively in clinical settings. Another important issue is the absorption rate of electromagnetic energy by the human body during the communication with BLE. For VNS devices, the absorption levels must not be harmful to the human bodies. By addressing those issues, the design of our proposed VNS prototype concerns the price range to be reasonable, the absorption rate to be safe, and the accessibility and longevity to be extended. We developed the prototype to increase the lifespan of the electroceutical to reduce the frequency of reimplantation, thus reducing the medical costs. By using a BLE-based communication app, the communication barrier between patients and doctors can be lowered, facilitating the patient follow-up studies [1,2,3,4,5,6,7,8,9]. The designed VNS prototype incorporates multiple layers of safety features to comply with FDA regulations and safety standards. Among these, the core is the thermal management system that prevents excessive heat accumulation during the recharging process. A series of thermal sensors embedded in the device case continuously monitor the temperature close to the skin surface. If the temperature exceeds a predefined safety limit, the charging is automatically paused. It only resumes when the device cools down to the safe level. To further enhance the safety, the device is encapsulated in biocompatible materials that protect sensitive internal components from moisture and physical damage. Additionally, the VNS operates within the frequency range approved by the international standards for medical devices, minimizing any interference with other electronic equipment to ensure safety within the human body. Even though wireless communication is the critical performance aspect, there are not enough studies that report the detailed development process for VNS or the improvement effort of short-range wireless communication, which may seriously hamper the usability and practicality of implantable electroceuticals. In our preliminary studies, we found that the communication distance of three meters for most VNSs should be extended for better usage of device in order to provide more freedom of movement for both patients and doctors or physicians. In fact, for the electroceutical implanted in the human body, an effective data communication can be the most important factor. If data communication fails, additional surgery must be conducted to either remove or replace the device from the patient. Therefore, it is crucial to have good communication. In this regard, we have set up several research goals and conducted a lengthy development procedure for a new VNS device with better communication performance. To verify the intended performance goals, we developed a new antenna and conducted an experiment using live animals to find out how the wireless communication range changes in a living implant environment. To be more precise, we set up four research goals: (1) it must be safe in the human body; (2) the output and intensity of communication waves must meet the standard (Figure 2 and Figure 3 verify the first and second goals, hence why no further explanation is given in this paper); (3) the unlicensed frequency of the Bluetooth band must be used for communication to increase the versatility; (4) a new antenna system for minimizing the communication attenuation effect caused by the living tissue in an actual living implant environment must be developed. For this goal, a receiving antenna suitable for implant is developed. To apply it to electroceuticals, the communication performance of the receiver antenna was checked, while the implantability and the electromagnetic wave safety was tested and verified. A steering algorithm and firmware were created to respond to changes in communication signals. We developed a low-power smart protocol for antenna control and tested the communication sensitivity for both transmission and reception distances before being introduced in the design. The transmission and reception distance target was set as seven meters, as previously mentioned. The communication distance was set based on the realistic consideration for the space size of operating room, hospital patient rooms, and treatment rooms. Finally, we conducted clinical trials on medium-sized animals using the newly developed electroceutical in real-life environmental tests [10,11].

## 2. Materials and Methods

This section illustrates the development process of the electroceutical prototype. The prototype has (1) the communication circuit and voltage output control module, (2) the real-time BLE communication processing algorithm, (3) the PoC application for parameter adjustment, (4) the exterior made of biocompatible materials, and (5) a wireless charging system for extended battery life. The main focus is to find a solution to reduce the communication attenuation. To achieve this, a receiving antenna suitable for implantation was developed. The communication performance of the receiver antenna was tested. Additionally, the implantability and electromagnetic wave safety of the electroceutical prototype were verified. In order to respond to changes in communication signals, a steering algorithm and firmware were developed and the communication sensitivity related to transmission/reception distance was verified. Various necessary sensors were integrated for various functions. The 7-m communication distance has to be achieved using the low-power Bluetooth technology, which should operate stably inside the human body. For this purpose, we conducted clinical trials on medium-sized live animals using the electroceutical prototype.

### 2.1. Development of Communication Module for BLE Performance Verification

This section first provides a review of wireless communication technology. Then, it shows our own developed received signal strength indicator (RSSI) module for testing the BLE performance. As shown in Table 3, the pros and cons of BLE, Zigbee, and MICS are outlined. Also, the application cases and the selection of appropriate technology were reviewed.

#### 2.1.1. Wireless Communication Technology Review

BLE is a technology that enables wireless communication between devices with a low power consumption. It is mainly used to transmit small amounts of data over a relatively short distance. The biggest advantage of BLE is its wide compatibility with most modern smart devices. Supported on smartphones, PCs, and tablets, BLE offers a long battery life due to the low power consumption. Due to these characteristics, BLE is widely applied to various consumer electronic products such as fitness trackers, smart watches, and home automation equipment. However, BLE may be unsuitable for applications requiring high bandwidth due to its limited communication range and data transfer speed. Zigbee is a low-power wireless network technology based on the IEEE802.15.4 standard [13]. It is mainly used in home automation, industrial control, and medical data collection. Zigbee’s main advantages are its scalability to build large device networks through multi-hop (mesh) networking and its ability to operate for a long period of time with low power consumption. Zigbee provides a reliable data transmission and strong security features. However, Zigbee networks can be complex to set up and manage, and compatibility issues can make device integration between manufacturers difficult. MICS is a wireless communication service designed for communications between medical implant devices. It enables an efficient wireless communication inside the human body using a dedicated frequency band (402–405 MHz). The main advantages of MICS are body-friendly communication and a low-power design to maximize the battery life of the implant. This technology is mainly used in medical implants such as heart pacemakers and cranial nerve stimulators, and can reliably transmit small amounts of data. However, MICS has the disadvantages of limited use, low data transfer rates, and the need for strict regulation and certification procedures as a medical device. Overall, the specific requirements of electroceuticals, such as communication range, data transfer rate, power consumption, network configuration complexity, and security requirements, must be carefully examined. These factors have a significant impact on the final technology selection and become decisive criteria for successful system implementation. Therefore, when choosing between BLE, Zigbee, and MICS, it is necessary to consider the characteristics of each technology and select the most suitable one for the given application and environment [11,12,13,14].

#### 2.1.2. Development of Wireless Transmission and Reception of RSSI Module

**Figure 4:**
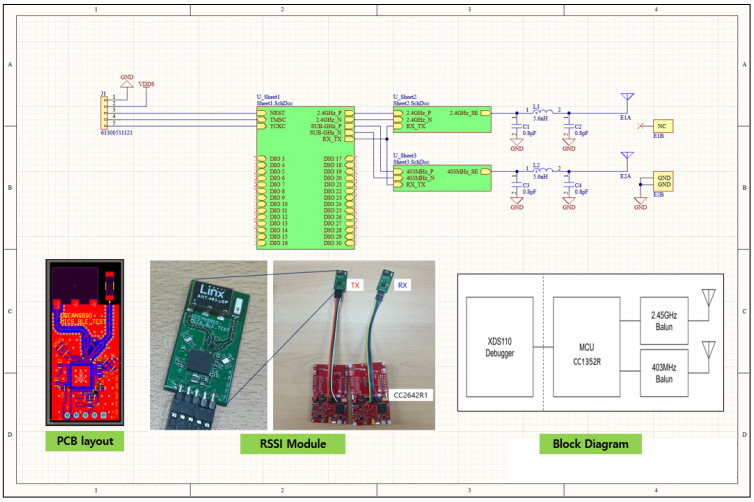
RSSI module schematic design and actual prototype module.

In this section, we elaborate on the development of our RSSI module to verify the BLE performance. In order to design a new RSSI module, a circuit block diagram was created. After selecting the MCU accordingly, the circuit and PCB design were conducted. The MCU is a CC1352R product from Texas Instrument (Dallas, TX, USA), and two 2.45 GHz and 403 MHz communication chips are Balun products from STMicroelectronics (Milano, Italy). With XDS110 Debugger (from Texas Instrument, Dallas, TX, USA) for programming and debugging. The MCU CC1352R from Texas Instrument (Dallas, TX, USA) with wireless communication function acts as a ‘brain’ where programs are stored and executed. It consists of a circuit for the 403 MHz frequency band. This is a part of an electronic device designed to process data according to algorithms stored in the MCU and convert that data into wireless signals to perform communication for a specific purpose. In addition, we produced a circuit module to measure this and prepared a CC2642R1 evaluation board from Texas Instrument (Dallas, TX, USA) for measurement and evaluation (Figure 4).

Communication sensitivity according to the measurement environment was checked using the developed RSSI module. After measuring within the air and saline solution, a plan was established to reflect the same conditions in the first animal clinical trial. The saline solution reflected the corresponding conditions with moisture similar to that of the human body, hence similar test conditions were established by implanting in a similar location where the actual electroceutical would be located in humans. The measurement distance was divided into 1-, 2-, and 3-m units. The basis for selecting the measurement distance is that most implantable medical devices currently guarantee a maximum communication distance of 3 m only, as shown in Table 4.

**Figure 5:**
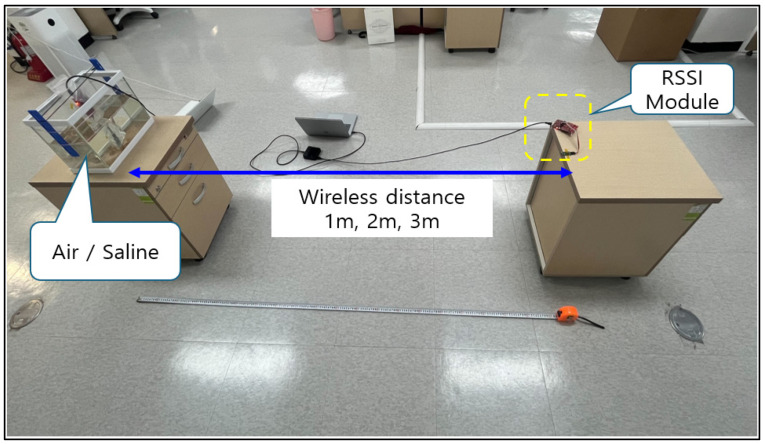
Air and saline measurement conditions.

Measurements were made indoors in both air and saline waters, while varying the distances (Figure 5). In Table 5, the measurement results show that BLE appears to be the best when compared with the results in air. The difference for each type appears to be negligible, and hovers around 10 dB. However, when a 5-m distance experiment was additionally conducted, no data could be received. For conditions in the saline water, any measurement values other than MICS could not be obtained properly. Prior to the first clinical animal trial, the communication attenuation in air and saline water was only compared and gauged qualitatively, hence there are no recorded numbers.

#### 2.1.3. First Animal Clinical Experiment Based on RSSI Module

**Figure 6:**
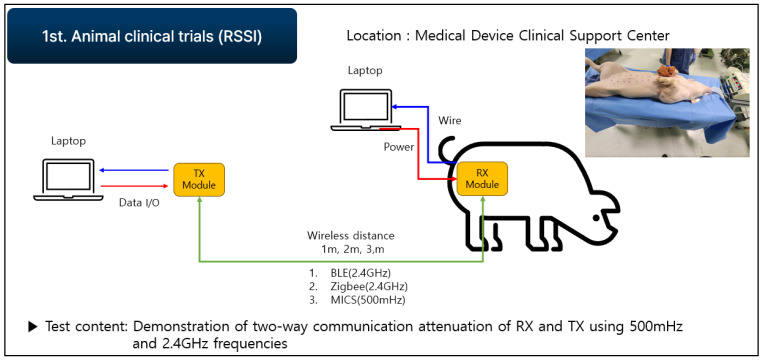
Communication attenuation experiment on live animals.

**Figure 7:**
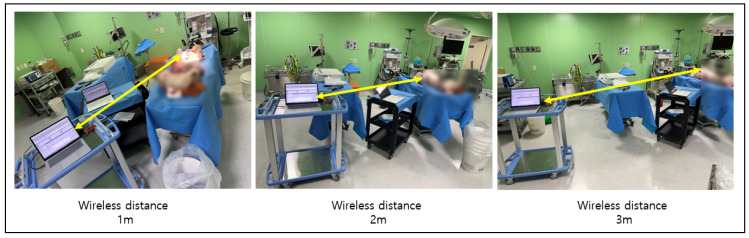
RSSI for clinical measurement conditions.

This section shows the experiment based on the results of previously conducted tests with a developed RSSI module to verify BLE performance. After establishing the experimental conditions and clinical test procedures for frequency communication attenuation verification by implanting the device in the animal body, clinical tests were conducted at the hospital’s preclinical center. The purpose is to verify the TX and RX two-way communication in the 500 MHz band (the MICS band) and the 2.4 GHz band (an unlicensed frequency band). The clinical procedure involved inserting a communication module into the live animal and connecting it to a measurement system in order to measure the signals (Figure 6 and Figure 7).

The RSSI measurement results showed no significant difference, regardless of the frequencies between 500 MHz and 2.4 GHz (Table 6). Even though there was no significant difference up to 3 m, the signal could not be detected at 5 m. Consequently, the signal could not be measured. Considering the adverse conditions of countless variables within the actual human body, it is important to maintain appropriate communication sensitivity even over a longer distance. To enable measurement at the final goal of 7 m, a secondary animal clinical test plan was established using the BLE frequency to reflect the electroceutical improvement solution at distances of 1, 3, 5, and 7 m.

### 2.2. New Electroceutical System with Improved Communication Performance

After verifying that the existing BLE communication distance was far short of the 7-m target, we developed a new electroweak system with improved performance to attain the goal.

**Figure 8:**
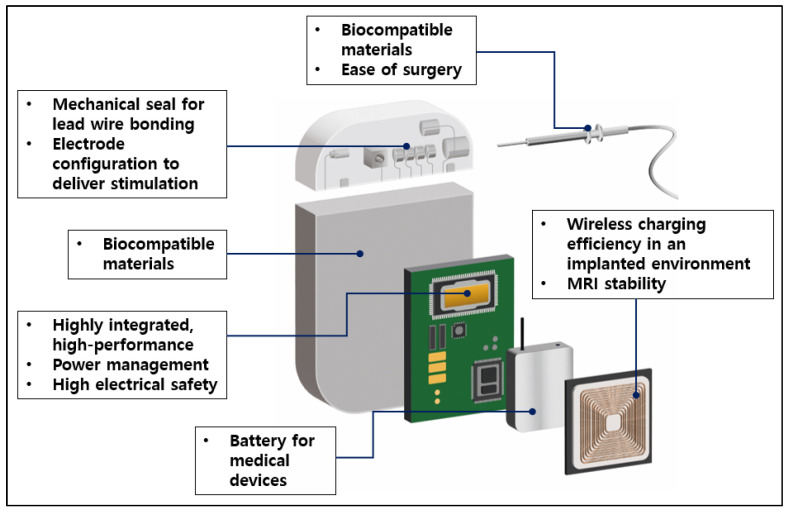
Structure of new electroceutical system.

The new electroceutical designed in this study is largely composed of three parts: the processor, controller, and energy source. Figure 8 shows the main components of the new electroceutical. The working part was implemented with the pulse generation function, which is a unique function of the electroceutical. The control unit uses low-power Bluetooth transmission and reception functions to ensure good communication. This increases usability over a wide and longer distance. This can actually become tailored for each patient’s situation in terms of a pulse protocol. The energy source is periodically charging with secondary batteries and uses the wireless charging method. Wireless charging will dramatically increase the lifespan of the electroceutical compared to primary batteries, thus extending the cycle of reoperation for patients [15,16,17,18].

The developed electroceutical charging system ensures a smooth and non-invasive charging process by using a hybrid process that utilizes both low-frequency magnetic induction and magnetic resonance. The external charger is designed to be user-friendly, allowing patients or caregivers to operate it with minimal instruction. To prevent any hazard, the system is equipped with multiple safety sensors that continuously monitor the skin temperature to prevent overheating. The sensors automatically shut off the power if the temperature reaches the safety threshold set by the FDA. In terms of size, the device measures 40.6 mm (width) × 60.6 mm (height) × 9.6 mm (depth). It is designed to be small and lightweight, weighing between 31–34 g. The small size as well as the smooth and rounded edges minimize the discomfort for both adult and pediatric patients, hence making the device ideal for long-term use. These features not only enhance the safety and efficacy of the device, but also ensure the compliance with the latest FDA regulations and medical standards. Additionally, we focus on extending the lifespan of the battery by using a wireless charging method with a secondary battery and aim for a lifespan of 10 years. Compared to previously released similar products, this will double the battery life if successful.

**Figure 9:**
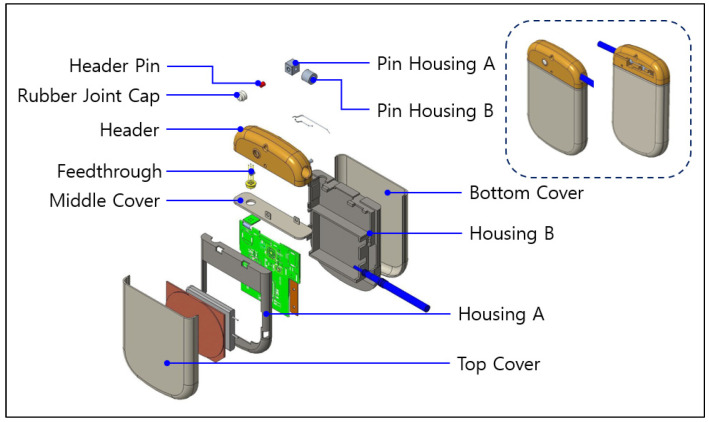
Exploded view of inner structure.

In the case of human implantable medical devices, parts must be made of biocompatible materials. The implant should not cause any problems when inserted into the human body. In this study, even from a prototype, having considered the animal preclinical trials, we used biocompatible materials, as shown in Table 7 and Figure 9. In addition, when inserted into the human body, a complete sealing was required to prevent any bodily fluid and blood from infiltrating the electroceutical. Thus, titanium parts were completely sealed using laser welding. Furthermore, waterproof, dustproof, and reliability were built into the design by separating the internal circuit and external contacts with a sealed connector.

**Figure 10:**
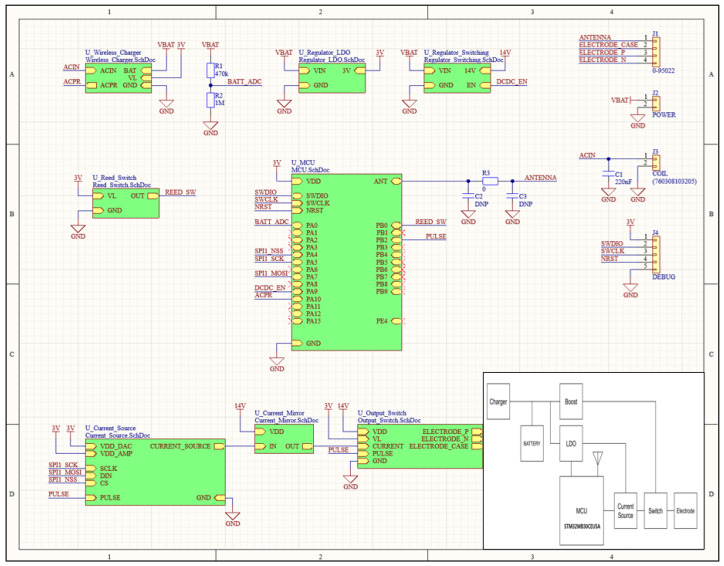
Prototype schematic design.

**Figure 11:**
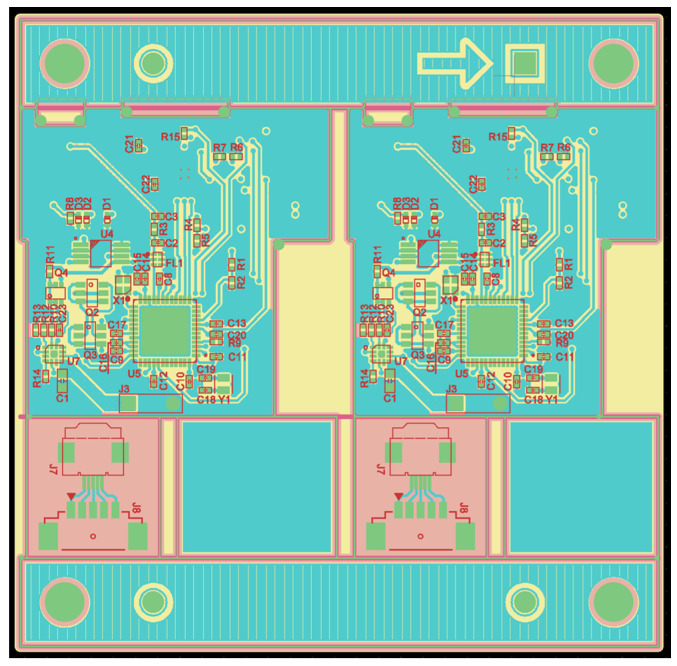
Prototype PCB layout.

The electroweak circuit and firmware design were conducted based on the circuit block diagram (Figure 10 and Figure 11). The flow of the developed algorithm is further explained in Appendix A. The energy stored in the battery is adjusted to the required voltage through a boost converter and LDO. The MCU uses this power to control the current source and provide a precise nerve stimulation through the electrodes by switching them on and off according to the programmed pattern. This process is mainly used in the medical field to manage pain or restore certain nerve functions, so the same process is reflected. The charger replenishes the battery by receiving energy from an external power source, and as a result, supplies the voltage and current suitable for the installed battery. The battery is a rechargeable cell for a prolonged life. Boost converter is used to increase the battery voltage. Typically, the battery voltage may be lower than the voltage required by the device, so this boost converter increases the voltage to ensure proper operation of the device. Low-dropout regulator (LDO) stabilizes voltage and reduces noise, and then receives the increased voltage from the boost converter and supplies a more stable voltage to the MCU. The MCU used here (STM32WB30CEU5A) is optimized for low-power Bluetooth models. The MCU controls the entire device according to our programmed algorithms, generates nerve impulse patterns, and communicates with other parts of the electroceutical.

The current source supplies a preset current to electrodes according to signals controlled by the MCU. This is used to generate a nerve stimulation. The switch is controlled by the MCU and turns the electrical connection between the current source and the electrode on and off as needed. This function allows the nerve stimulation to occur only at a specific time. Electrodes are, in general, used for therapeutic purposes by delivering electric current to specific areas of the body to stimulate them. Therefore, that is the key function for the electroceutical when prescribed by a doctor.

**Figure 12:**
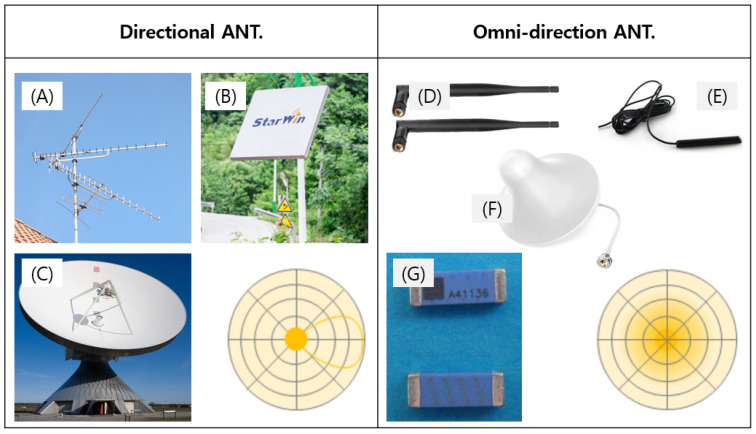
Comparison between directional antenna and omni-direction antenna: (**A**) yagi antenna, (**B**) panel antenna, (**C**) dish antenna, (**D**) rod antenna, (**E**) rubber duck antenna, (**F**) dome antenna, (**G**) chip antenna.

**Figure 13:**
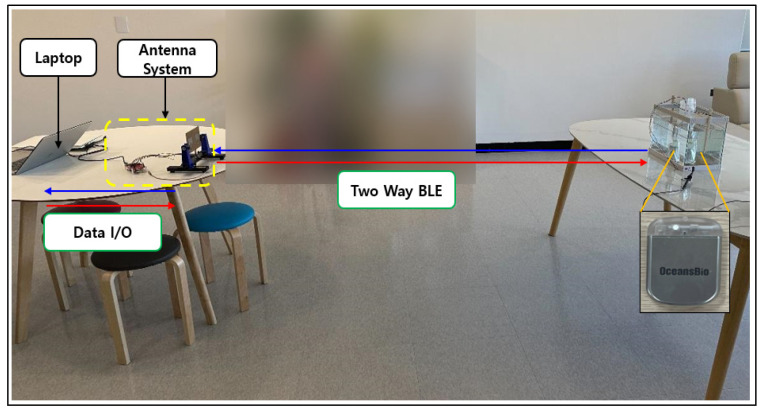
Newly developed PAA control system.

**Figure 14:**
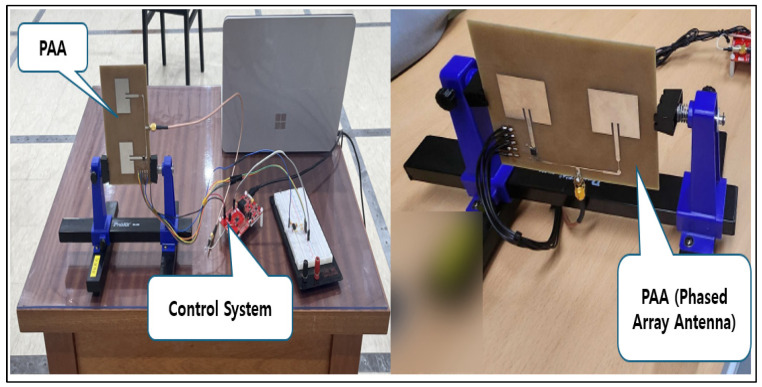
Testing of newly developed PAA.

To verify the BLE performance, we initially selected a chip antenna, which is a type of non-directional antenna mounted on a circuit. This non-directional antenna is mainly used for mobile phones or portable devices. As shown in Figure 12, for this type of antenna communication is difficult and the signal is weak in certain directions, so it is widely used for general purposes. However, since the 7-m communication distance target of our VNS could not be reached, it was necessary to change the antenna. Therefore, we developed a new directional antenna for the prototype. There are many directional antenna products in the market, but most of them are high-output, high-voltage products. Due to the safety requirement of the VNS, the product must operate at a low power. So, it was crucial to develop a directional antenna suitable for a BLE-based electromagnetic system. The developed BLE, phased array antenna (PAA), strengthens the received signal by forming an optimal beam, depending on the position of the electroceutical (Figure 13 and Figure 14). For this purpose, the PAA transmission antenna gain was set to 5 dB to compensate for the communication signal attenuation, caused by the insertion of electroweak into the human body. In addition, the optimal beam search time was set to be 5 s to quickly form an optimal beam path according to the movement of the electroceutical, thereby obtaining the desired communication signal. It should be noted that the elaborations on the detailed developmental process of PAA is not given here. It is due to the fact that the details are very long and technical, so only the brief outline regarding the rationale for selecting the PAA and the other issue are provided in this section.

**Figure 15:**
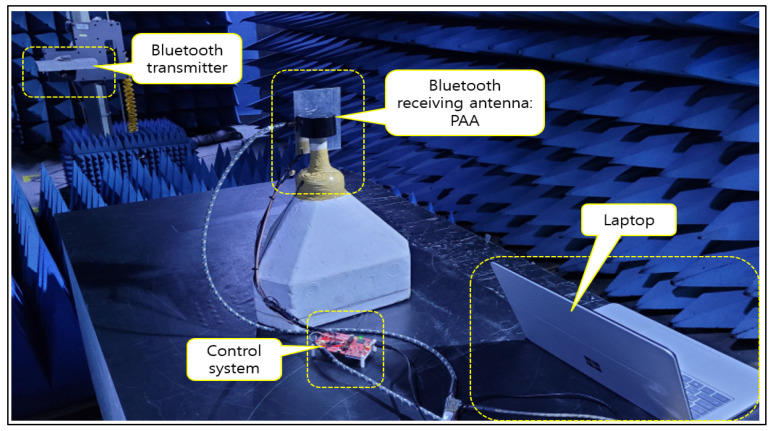
RF signal input and beam steering test.

**Figure 16:**
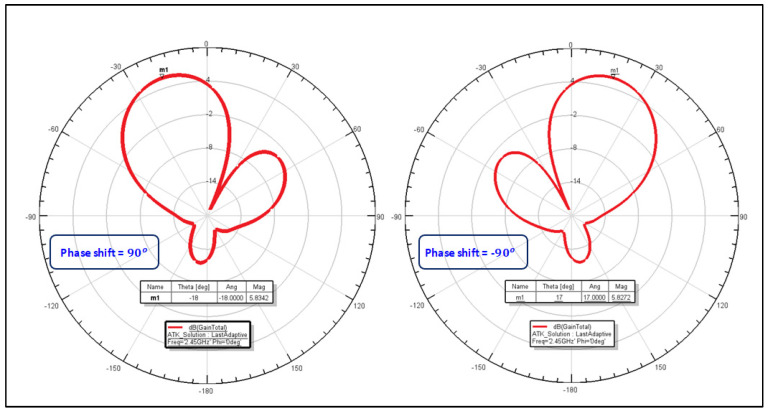
Beam steering for +/−90 degrees of phase shift.

In the radio frequency test lab, it was confirmed that the RF signal passes through a directional coupler with the Bluetooth transmitter and receiver (Figure 15). The verification of the antenna beam steering direction was conducted without any problem (Figure 16). With a phase shift of ±90 degrees, the main beam could be steered appropriately by the PAA within an angle of ±18 degrees, hence demonstrating its directional operation functionality.

## 3. Results and Discussion

**Figure 17:**
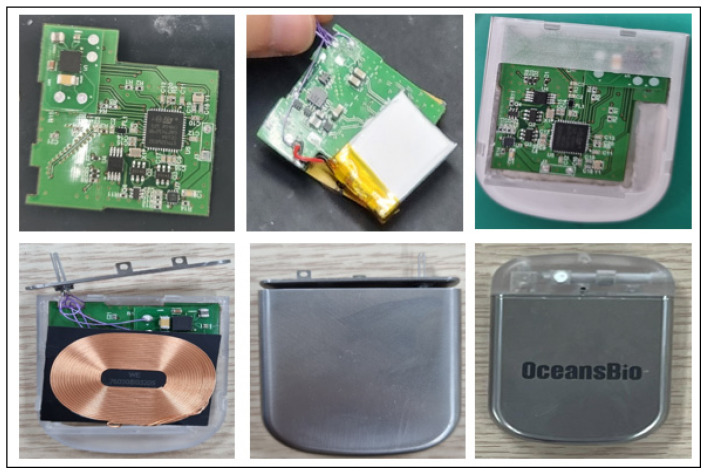
Prototype manufacturing and assembly.

In this section, we present a detailed aspect of the new prototype based on the results obtained through the preliminary development of the RSSI module and the first animal clinical test. This prototype encompasses a human-safe BLE communication that is equipped with the directional antenna with a distance of seven meters, the communication attenuation algorithm to improve the communication performance, and the housing parts and electronic circuit design that reflect biocompatible materials. As shown in Figure 17, new prototype parts were manufactured and assembled.

**Figure 18:**
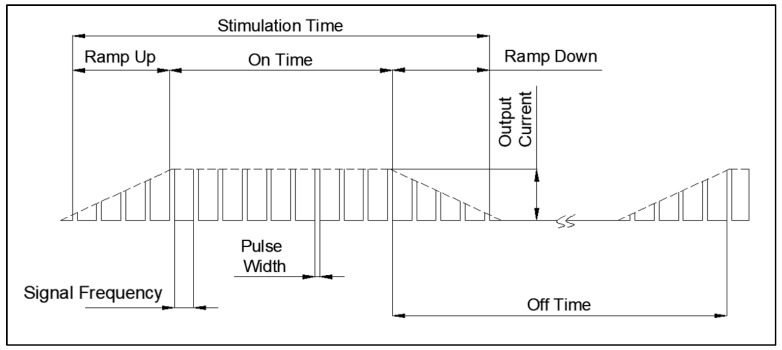
VNS stimulation waveform.

The VNS electroceutical delivers a series of stimulation pulses with the programmed frequency, amplitude, pulse width, and duty cycle (Figure 18). At frequencies above 10 Hz, the output current increases progressively across Ramp Up to the programmed output current at the beginning of each stimulus pulse train. It then decreases from the programmed output current to zero across Ramp Down towards the end of each [19]. The electroceutical current output measurement formula is as follows.

I = V/R(1)

Here, I stands for current (A), V stands for voltage (V), and R stands for resistance (Ω). For example, if the output voltage of the electroceutical is 12 V and the output resistance is 3 Ω, the current is as in Equation (2).

I = 12 V/3 Ω = 4 A(2)

The main function of the electroceutical is to connect a microcurrent to the vagus nerve and stimulate it. The main parameters of our prototype was compared as in Table 8, to verfy that all the main performance numbers are up to the standards. The comparion was using the Livanova VNC, which is currently the most widely adpted product worldwide. As a result of the test, it was confirmed that the parameter values and output values were equivalent to the global benchmark levels. The prototype’s adjustable parameters allow the user or medical professionals to optimize the device performance for specific treatment goals. Table 9, Table 10, Table 11 and Table 12 show the parameter measurements of our prototype compared to those of Livanova VNC [20,21].

### Verification of Prototype with Second Animal Clinical Trial

**Figure 19:**
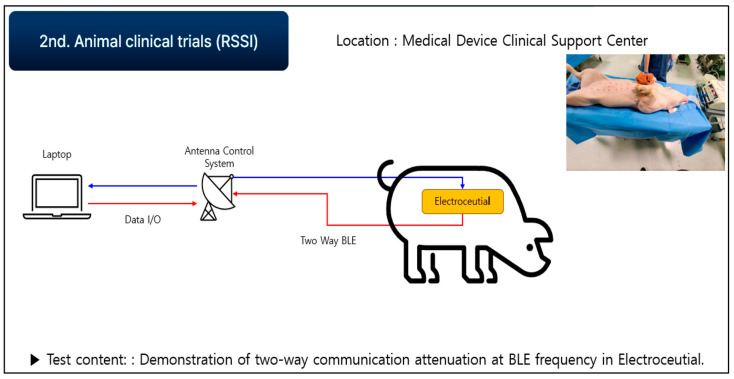
Communication attenuation measurement for electroceutical.

**Figure 20:**
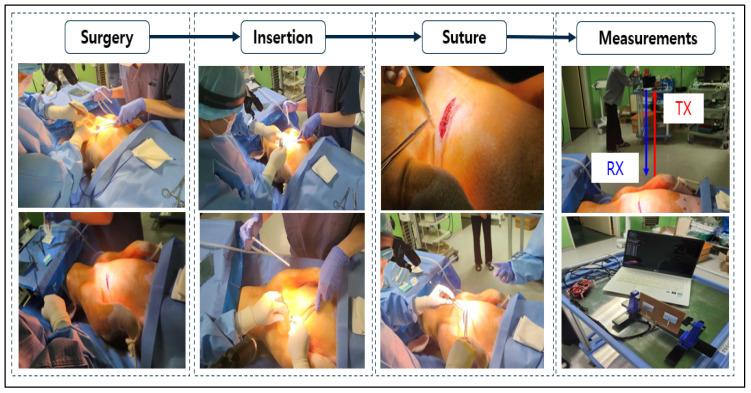
Second clinical trial procedure for electroceutical.

In order to confirm that a smooth communication is possible at a distance of seven meters using our prototype, the second round of live animal clinical trials were conducted. Communication attenuation was measured using a live pig, as shown in Figure 19 and Figure 20, and verified by analyzing the collected data. In the second test, the electroceutical prototype was installed inside the pig’s body. The insertion was performed in the same location as the humans.

In this experiment, we checked the communication attenuation performance along the preset distances using the developed prototype (Table 13). We confirmed that a smooth communication was achieved at the target distance of seven meters. The directional antenna (PAA Ant.) showed a good performance, displaying no significant drop in communication from one to five meters. From a distance of seven meters, the signal became weaker, yet it was still able to communicate with external devices. It seems that there is no significant hindrance due to the occurrence of radio wave interference, even when residing inside the body. Additionally, we tested the performance of conventional non-directional antenna from a distance of seven meters. The non-directional antenna (Chip Ant.) in the figure performed poorly and was unable to establish any communication with external devices at this distance. However, this was anticipated. In this light, our prototype showed a much wider range than the currently commercially available VNS products. This aspect can provide patients, doctors, and physicians an enhanced capability in terms of being able to overcome space constraints commonly found in large-scale, highly crowded hospitals in South Korea (or any other countries).

**Figure 21:**
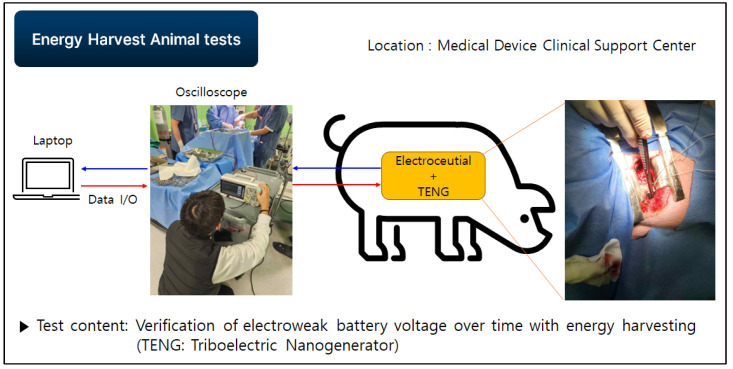
Energy harvest experimentation for prototype.

Figure 21 shows the energy harvesting test for our prototype during the animal clinical experimentation. The test measures the change in battery voltage in relation to the application of a triboelectric generator (TENG). The main purpose is to increase the lifespan of VNS batteries. TENG is a new type of power generation device that works by producing electricity through friction between different materials. It is a new technology that is expected to play an important role in the future. In testing the energy harvesting with our prototype, a minute amount of voltage was observed to be generated. However, it could not reach the practical level, and further studies are needed in order to apply the technology for our final product.

**Figure 22:**
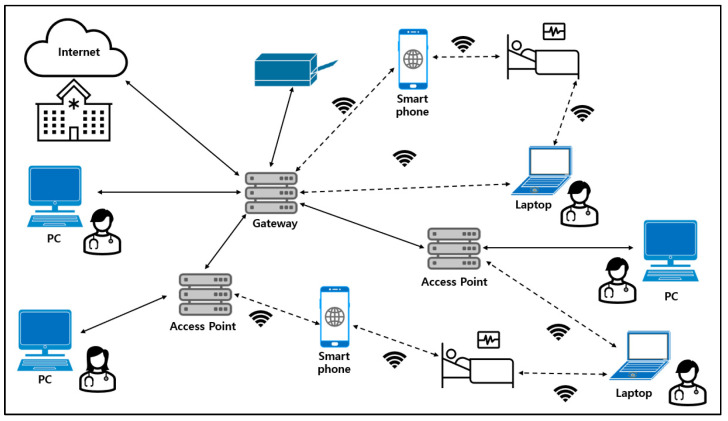
Hospital with 2.4 GHz frequency-based IoT.

The prototype electroceutical developed in this study has several advantages. (1) Surgical environment: in the operating room, lots of medical equipment and medical staff are complexly intertwined. Therefore, the conventional three-meter restrictions can be quite cumbersome and difficult to manage in such an environment. The prototype electroceutical enables a smooth BLE communication from a longer distance, even in these adverse conditions. (2) Hospitalization environment: even when a patient is lying alone in a hospital room, the hospital’s IoT system can be connected through a mobile phone application, allowing patients to receive the VNS prescriptions within the room without having to travel to the doctor’s office. This can actually make a much safer environment for patients, especially when they have just finished an operation or surgery to install the VNS. (3) Patient benefits: even when patients are in their own home, the caregiver or patient themselves can access the VNS remotely from a much longer distance. This allows the person with the VNS to not move around frequently in order to find the communication range [21,22,23,24,25,26,27,28,29]. Our presented approach can effectively improve the communication function of implantable medical devices, increase the efficiency of medical devices, and provide new opportunities to improve the quality of life for patients. In particular, it has the potential to improve treatment outcomes by providing customized treatment methods to patients with various neurological diseases such as epilepsy and depression. As shown in Figure 22, it is perceivable that the development and use of implantable medical devices such as the electroceutical will play an important role in future medicine [30,31,32,33,34].

## 4. Conclusions

This paper developed a new VNS electroceutical system through an innovative approach to overcome the communication limitations of existing implantable devices. A new PAA with a better communication performance was developed and applied to the prototype. In order to effectively respond to changes in communication signals, we developed a steering algorithm and firmware, and designed a smart communication protocol that operates at a low power that is safe for patients. This protocol is intended to improve communication sensitivity related to the transmission and reception distance and has been integrated into the electroceutical prototype through the integration of sensors. Based on this technical approach, the heightened effectiveness as well as the safety of the prototype have been verified with the help of two sets of live animal clinical tests. We confirmed the communication attenuation performance and also confirmed that a smooth communication was achieved even at a distance of seven meters. The performance of the directional antenna (PAA) mounted on the prototype was evaluated and compared with the previously used omni-directional antenna. As a result, the omni-directional antenna was unable to communicate from a distance of seven meters, meaning that our prototype has a much wider communication range. Such a performance represents a new heightend exendability, a wider applicability, as well as a much easier way to use the device for patients, doctors, and physicians in crowded and congested hospital spaces. For example, in an operating room, a lot of medical equipment and medical staff are intricately intertwined. Our prototype enables a smooth BLE communication, even in these adverse conditions. In addition, our prototype allows patients to receive VNS prescriptions within the hospital room without having to travel to the doctor’s office. This makes for a much safer environment for patients, especially when they have just finished an operation or surgery for VNS. The new electroceutical can increase opportunities to receive remote prescriptions for patients from outside the hospitals with less space constraint.

The new device has also been developed with the goal of enhanced safety for the user. For example, the charging method applied is not the simple electronic induction used in typical smartphones, nor is it intended for rapid charging. The device represents the fusion of electronic induction and magnetic resonance methods. Since the electronic device is implanted in the body, the wireless charging must consider the thickness of the dermal layer (typically 25 mm). If the transmission goes directly from the inside of the titanium cover to the outside, wireless charging is not possible. Therefore, the header part of the electroceutical is made of plastic material, which allows for the transmission of signals to the outside. This effectively enables the wireless charging. During the charging, the temperature change for FDA-approved implantable devices should be below two degrees Celsius. This has been the target for our new product, and so far, there have been no particular issues. Another important aspect of our prototype is the potential application of energy harvesting to the device. We have tested the energy harvesting for our prototype during the animal clinical experimentation. The test measures the changes in battery voltage in relation to the application of TENG. The main purpose is to increase the lifespan of VNS batteries. Even though it was observed that a minute amount of voltage can be generated, it could not reach the practical level. Further studies are needed in order to apply the technology for our final product. In conclulsion, the results of this study have laid an important foundation for solving the communication problem of implantable medical devices in the human body and show that it can be used as a solution to improve the communication attenuation problems in the development of not only electroceutical but also other implantable products in the future.

## Figures and Tables

**Table 1:** Detailed information on different types of electroceuticals.

Company	Electroceutical	Model	Figure	Size	Weight	Life	RC	Wireless
Livanova (Houston, TX, USA)	VNS	AspireSR (MODEL 106)	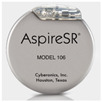	52 × 52 × 6.9	25	1–16 year Up to setting	X	BLE
		SenTiva (MODEL 1000)	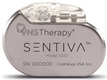	15 × 32 × 6.9	16		X	BLE
Abbott (Chicago, IL, USA)	SCS	Eterna	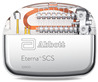	10.7 × 4.3 × 3	26.4	1–10 year Up to setting	O	BLE
		Proclaim	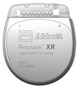	55.5 × 49.5 × 13.4	48.9		X	BLE
	DBS	Infinity	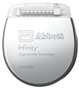	55.5 × 49.5 × 13.4	48.9		X	BLE
Boston Scientific (Marlborough, MA, USA)	DBS	Vercise Genus™ DBS System	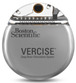	55 × 45 × 11	N/A	1–15 year Up to setting	O	BLE
			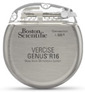	52.1 × 46 × 10.7	N/A		O	BLE
			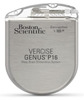	72 × 49.6 × 11.6	N/A		X	BLE

**Table 2:** Frequency status of major global products.

Company	Model	Frequency	Ouput	Wireless	Certification
Boston Scientific (Marlborough, MA, USA)	DB-1432	2.402–2.48 GHz	2.1 mW	BLE	28 April 2020
Medtronic (Dublin, Ireland)	Cobal XT VR MRI SureScan	2.402–2.48 GHz	1 mW	BLE	2 April 2020
Abbot (Chcago, IL, USA)	32400	2.402–2.48 GHz	2 mW	BLE	25 October 2022
Axonics (Irvine, CA, USA)	1101	402–405 MHz	0.0002 mW	MICS	23 September 2019
Nevro (Redwood City, CA, USA)	IPG3000	2.402–2.48 GHz	2.7 mW	BLE	19 May 2022
Impulse Dynamics (Mariton, NJ, USA)	CCMX11	402–405 MHz	0.0008 mW	MICS	5 May 2021
Canary Medical (Carlsbad, CA, USA)	CTE1	402–405 MHz	0.0095 mW	MICS	30 July 2021
Saluda Medical (Bloomington, MN, USA)	3042	402–405 MHz	0.0004 mW	MICS	23 May 2022
Medtronic (Dublin, Ireland)	37612	175 KHz	N/A	ULP-AMI	25 May 2018

**Table 3:** Comparison between major wireless communication technologies.

Contents	BLE	Zigbee	MICS
Power consumption	Low Below 10 mW	Low Below 10 mW	Very low Below 1 mW
Data transfer speed	Fast 1 Mbps	Slow 250 kbps	Very slow 40 kbps
Networking	Star	Mesh	Point to point
Security	Medium	High	High
Price	Cheap	Cheap	Expensive
Application	Wearable device Smart home beacon	Home automation Industrial automation Light control	Medical implant device
Advantages	Fast data transfer speed Low price	Mesh networking High security	Very low power consumption Very high security
Disadvantages	Short communication distance Non-mesh networking	Slow data transfer speed	High price Complex setup

Select guide. 1. When fast data transfer speeds are important: BLE. 2. When mesh networking is needed: Zigbee. 3. When low power consumption and high security are important: MICS.

**Table 4:** Guaranteed communication distance for major products.

**Manufacturer:**	Boston Scientific (Marlborough, MA, USA)	Abbott (Chicago, IL, USA)	Nevro (Redwood City, CA, USA)
**Distance:**	3 m	2 m	1.5 m

**Table 5:** RSSI values from measurement (dB).

Type	Condition	1 m	2 m	3 m	5 m
MICS	Air	−69.8	−71.4	−74	N/A
BLE	Air	−59.4	−65.8	−56.4	N/A
Zigbee	Air	−61	−57	−64.2	N/A
MICS	Saline	−75.5	−81.5	−80.5	N/A
BLE	Saline	−83.3	N/A	N/A	N/A
Zigbee	Saline	N/A	N/A	N/A	N/A

**Table 6:** RSSI values from clinical measurement (dB).

Contents	Condition	1 m	2 m	3 m	5 m
MICS	Clinical	−67.9	−60.1	−61.9	N/A
BLE	Clinical	−64.2	−55	−69.3	N/A
Zigbee	Clinical	−52.2	−63	−54	N/A

**Table 7:** Main part composition for biocompatibility.

Part	Raw Material	Amount	Note
Top Cover	Titanum	100%	Grade 2
Middle Cover	Titanum	100%	Grade 2
Bottom Cover	Titanum	100%	-
Housing A	PC	100%	-
Housing B	PC	100%	-
Feedthrough	Platinum-iridium alloy	N/A	Grade 2
Header Pin	SUS316L	100%	-
Pin Housing A	SUS316L	100%	-
Pin Housing B	SUS316L	100%	-
Header	Epoxy	100%	-
Rubber Joint Cap	Silicone	100%	-

**Table 8:** Parameter specification.

Livanova Model 106 Parameter			Prototype Parameter	
Current (mA)	0–3.5	0.0–2.0/0.125 Steps	0–3.5	0.0–2.0/0.125 Scale
		2.0–3.5/0.250 Steps		2.0–3.5/0.250 Scale
Pulse Width (μsec)	130–1000	130–250–500–750–1000	130–1000	130–250–500–750–1000
Frequency (Hz)	1–30	1–2–5–10–15–20–30	1–30	1–2–5–10–15–20–30
On Time (sec)	7–60	7–14–21–30–60	7–60	7–14–21–30–60
Off Time (min)	0.2–180	0.2–0.3–0.5–0.8–1.1–1.8–3	0.2–180	0.2–0.3–0.5–0.8–1.1–1.8–3
		5–60/5 Scale		5–60/5 Scale
		60–180/30 Scale		60–180/30 Scale

**Table 9:** Example of parameter current.

Current		Model 106	Prototype
Unit: mA		Load: 3 K	Load: 3 K
0.0–2.0 /0.125 Steps	2	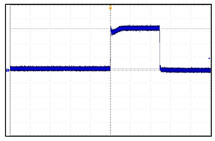	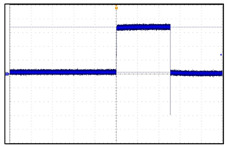

**Table 10:** Example of parameter width.

Width	Model 106	Prototype
Unit: μA	Load: 3 K	Load: 3 K
130	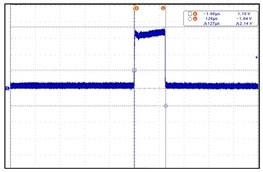	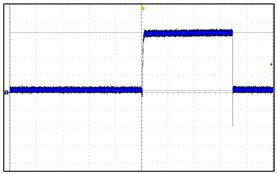

**Table 11:** Example of parameter frequency.

Frequency	Model 106	Prototype
Hz	Load: 3 K	Load: 3 K
1	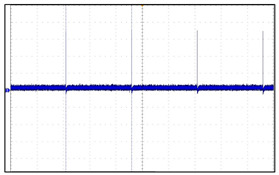	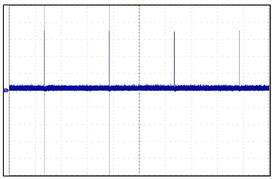

**Table 12:** Example of parameter on time.

On Time	Model 106	Prototype
Sec	Load: 3 K	Load: 3 K
21	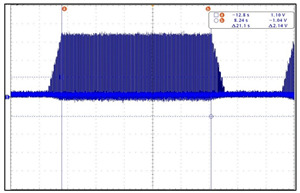	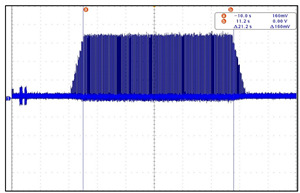

**Table 13:** RSSI clinical measurements for our prototype electroceutical.

Distance	1 m (PAA Ant.)	3 m (PAA Ant.)	5 m (PAA Ant.)	7 m (PAA Ant.)	7 m (Chip Ant.)
RSSI (dB)	−68 dB	−78 dB	−78 dB	−84 dB	−93 dB
Clinical Details	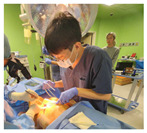	** 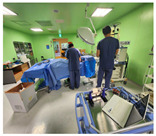 **	** 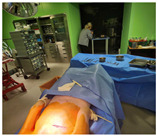 **	** 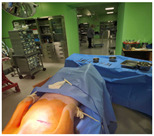 **	** 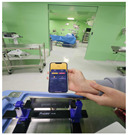 **

## Data Availability

Anyone interested in our experimental data should contact the corresponding author via the email.

## Appendix A

**Algorithm A1.** Control Protocol
static void Pulse_Start(void) { HW_TS_Stop(TimerId.Pulse); Pulse.State = ENABLE; Pulse.Count = 0; Pulse.Period = 0; // In the Pulse_Start function, set when the pulse starts and call the HW_TS_Start function to start the timer. // The Pulse_Task function performs different operations depending on the pulse mode status. HAL_GPIO_WritePin(DCDC_EN_GPIO_Port, DCDC_EN_Pin, SET); MAX5531_Command(0b1111, 0); MAX5531_Command(0b1100, 0×0400); //Enter Standby, Set RefV: 1.940 V switch (Pulse.Mode) { case NORMAL: { HW_TS_Start(TimerId.Pulse, 1×1000×1000/CFG_TS_TICK_VAL/Param_Table.Frequency[Normal_Param.Frequency]); break; } case MAGNET: { HW_TS_Start(TimerId.Pulse, 1×1000×1000/CFG_TS_TICK_VAL/Param_Table.Frequency[Magnet_Param.Frequency]); break; } default: break; } } static void Pulse_Task(void) { switch (Pulse.Mode) { case NORMAL: { switch (Pulse.Ramp) { case RAMP_DISABLE: { MAX5531_Command(0b1111, Param_Table.Current [Normal_Param.Current]);
